# Homogenization of Thermal Properties in Metaplates

**DOI:** 10.3390/ma17184557

**Published:** 2024-09-17

**Authors:** David Faraci, Claudia Comi

**Affiliations:** Department of Civil and Environemental Engineering, Politecnico di Milano, Piazza Leonardo da Vinci 32, 20133 Milan, Italy; claudia.comi@polimi.it

**Keywords:** homogenization, asymptotic homogenization, thermal expansion, thermoelasticity, metamaterial, metaplate

## Abstract

Three-dimensional metamaterials endowed with two-dimensional in-plane periodicity exhibit peculiar thermoelastic behaviour when heated or cooled. By proper design of the unit cell, the equivalent thermal expansion coefficient can be programmed and can also reach negative values. The heterogeneity in the third direction of such metamaterials also causes, in general, a thermal-induced deflection. The prediction of the equivalent thermal properties is important to design the metamaterial suitable for a specific application. Under the hypothesis of small thickness with respect to the global in-plane dimensions, we make use of asymptotic homogenization to describe the thermoelastic behaviour of these metamaterials as that of an equivalent homogenous plate. The method provides explicit expressions for the effective thermal properties, which allow for a cost-effective prediction of the thermoelastic response of these metaplates.

## 1. Introduction

Thermoelastic metamaterials are an emerging class of architectured materials, typically constructed by the periodic repetition of a unit cell, which is designed to combine the thermal and elastic properties of the constituent materials to obtain peculiar behaviours. Natural materials usually exhibit a positive coefficient of thermal expansion (CTE) with few exceptions like, e.g., zirconium tungstate [1] and zirconium pyrovanadate [2] that are endowed with a negative CTE. However, the use of these latter materials for engineering applications is limited due to their low mechanical properties [3]. Composite materials and metamaterials can be designed to have negative or zero CTE [4] and good mechanical performances, thus attracting a lot of interest in the recent literature. Metamaterials with different cell geometries have been proposed in the literature to obtain isotropic [5], anisotropic [6] and auxetic [7] metamaterials with negative CTE. The proposed cells can have a generic three-dimensional geometry and, in that case, the metamaterial is obtained by their periodic repetition in three dimensions, or the cell can have a heterogeneous in-plane design, extruded in the third direction. In that case, the metamaterial (or metaplate) is obtained by two-dimensional repetition of the cell.

These typical geometries employed in the available literature are not suitable to be fabricated by lithographic processes commonly employed in Micro-Electro-Mechanical systems (MEMS) [8]. A new metamaterial with programmable CTE (positive, null or negative), fully compatible with microlithography, has been recently proposed [9]. It consists of the two-dimensional repetition of a star-shaped unit cell with a first structural layer made in silicon and a partial metallization, e.g., of nickel, on the top of some portions of the base silicon structure. The non-symmetrical layered configuration causes, in general, a thermal-induced deflection upon heated that, with a proper design of the unit cell, can be tuned (upward, downward or to zero).

Finite element analyses of such three-dimensional metamaterials can have a large computational burden, especially when the number of cells is huge or the unit cell geometry is complex and requires a fine mesh. Asymptotic homogenization is a mathematical technique that can reduce the computational cost of numerical analyses, see, e.g., the fundamental books [10,11] on the topic. For linear problems, the method provides an explicit expression for the effective homogenized properties, allowing for the description of a complex metamaterial as an equivalent homogenous body. In the literature, such a technique has been employed for the characterization of periodic solids in elasto-dynamic, e.g., for composite materials [12], locally resonant materials [13] and at high frequencies [14]. The same approach has been successfully applied to multi-physics problems, such as in linear [15] and non-standard [16] thermoelasticity, as well as to many other coupled problems [17].

A three-dimensional periodic medium with a two-dimensional periodicity, e.g., the one proposed in [9], can be homogenized to obtain an equivalent plate if the transversal dimension is small with respect to the in-plane dimension of the body. If the unit cell of the periodic body can be modelled as a plate, the homogenization procedure can be performed starting from the governing equations of structural plate theories. This has been carried out in the linear regime for perforated [18], composite [19] and locally resonant [20] metamaterial plates, as well as for the nonlinear theory proposed by Föppl and von Kármán [21,22]. When the behaviour of the single unit cell cannot be described by the plate theory, one must start from the three-dimensional formulation of continua and, fixing the ratio between the unit cell height *h* and its in-plane dimension *ℓ*, perform the asymptotic study of the problem as ℓ→0 [23,24]. In [25], the case where ℓ→0 first and then h→0, and vice versa, are also considered showing that different homogenized properties are obtained in the three limit cases.

In this work, we focus on the thermoelastic homogenization of a three-dimensional medium endowed with a two-dimensional periodicity. Starting from the method proposed in [26], where the focus is on the evaluation of the effective plate stiffness only, we extend the asymptotic study to also account for the thermal behaviour of the constituent materials. The new homogenization scheme of the thermoelastic metaplate is then implemented and its effectiveness is shown with some numerical examples.

This work is organized as follows: after the present introduction, the hypotheses and the governing equation of the problem are stated in Section 2. Then, in Section 3, a two-scale asymptotic homogenization procedure is developed and the effective properties of the thermoelastic metaplate are provided. Some remarks on the homogenization technique are presented in Section 4. Section 5 presents the results that can be obtained by the new proposed homogenization approach with reference to two metamaterials: one with a negative equivalent CTE and the other with a positive equivalent CTE. A discussion about the proposed method is carried out in Section 6 while conclusions are given in Section 7.

*Notation*: Latin indices (i,j,…) run from 1 to 3 while Greek indices (α,β,…) run from 1 to 2. Summation over repeated subscript and superscript in a term or a product is always implicitly assumed. The unit vector of the i-th axis is indicated with $e_{i}$, while the components of the second-order identity tensor are indicated with $\delta_{ij}$. Partial derivates with respect to the spatial variable $x_{i}$ and the fast spatial variable $y_{i}$ are denoted, respectively, by a comma and a vertical bar:

(1) $${\left(⋄\right)}_{,i}=\frac{\partial (⋄)}{\partial x_{i}}\;\mathrm{and}\;{\left(⋄\right)}_{|i}=\frac{\partial (⋄)}{\partial y_{i}}.$$

## 2. Problem Definition

**Figure 1:**
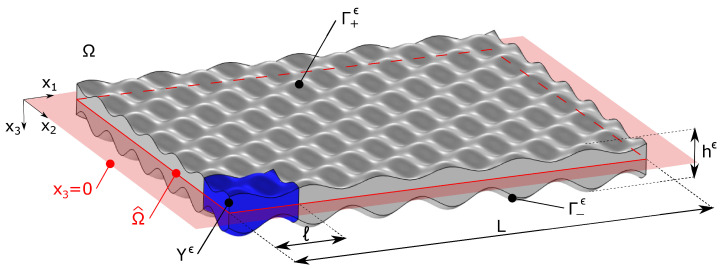
Geometry of the three-dimensional solid Ω with a two-dimensional periodicity in the plane $x_{3}=0$ (in red). The unit cell $Y^{\epsilon }$ is highlighted in blue.

Let us consider a generic three-dimensional solid Ω, referred to a coordinate system $x=x_{i}e_{i}$ as shown by the sketch of Figure 1, which is characterized by a two-dimensional ($x_{1}-x_{2}$) periodicity. Such a metamaterial is obtained by the repetition of a general three-dimensional unit cell $Y^{\epsilon }$ (highlighted in blue) that is periodically translated with a translation vector belonging to the reference plane $x_{3}=0$ (in red). Such a unit cell, which may have a varying out-of-plane thickness, can be made of one or more component materials and can include voids. The intersection of the solid Ω with $x_{3}=0$ is denoted by $\hat{\Omega }$ and its characteristic length by *L*. In the $x_{3}$-direction, the body is bounded by two surfaces $\Gamma_{+}^{\epsilon }$ and $\Gamma_{-}^{\epsilon }$ of equations $x_{3}=h_{+}^{\epsilon }$($\hat{x}$) and $x_{3}=h_{-}^{\epsilon }$($\hat{x}$) (respectively), where $\hat{x}$$=x_{\alpha }$$e_{\alpha }$ denotes the in-plane position vector. Note that, in general, the metamaterial plate is non-symmetric with respect to its mid-plane.

We assume the following hypotheses:

1. the characteristic in-plane dimension *ℓ* of the unit cell $Y^{\epsilon }$ is much smaller than the characteristic in-plane dimension of the periodic media, i.e., ϵ=ℓ/L≪1;
2. the maximum transversal dimension $h^{\epsilon }$ of the body is much smaller than its characteristic in-plane dimension, i.e., $h^{\epsilon }=\max_{\hat{x}\in \hat{\Omega }}\left\{h_{+}^{\epsilon }\left(\hat{x}\right)-h_{-}^{\epsilon }\left(\hat{x}\right)\right\}\ll L$;
3. external loadings and temperature variations are quasi-statically applied so that transient effects can be neglected;
4. temperature variations are sufficiently small to assume that all material properties can be considered temperature independent.

The first two assumptions allow us to perform an asymptotic analysis as ϵ→0, for a fixed ratio $h^{\epsilon }/\ell$, of the real three-dimensional metamaterial (endowed with a two-dimensional periodicity) to describe its thermoelastic behaviour as that of an equivalent homogenous two-dimensional plate of mid-surface $\hat{\Omega }$.

Neglecting transient effects, as assumed in the third hypothesis, the steady-state thermoelastic problem for a three-dimensional continuum, see [27,28], is governed by the equations

(2) $$\left\{\begin{array}{cc} \sigma_{ij,j}^{\epsilon }+F_{i}^{\epsilon }=0 & \mathrm{in}\;\Omega \\ q_{i,i}^{\epsilon }=r^{\epsilon } & \mathrm{in}\;\Omega \end{array}\right.\;,$$

where $\sigma_{ij}^{\epsilon }=\sigma_{ij}^{\epsilon }\left(x\right)$ is Cauchy’s stress tensor, $F_{i}^{\epsilon }=F_{i}^{\epsilon }\left(x\right)$ is the volumetric body force, $q_{i}^{\epsilon }=q_{i}^{\epsilon }\left(x\right)$ is the heat flux and $r^{\epsilon }=r^{\epsilon }\left(x\right)$ is the internal heat production. Under the hypothesis of linear behaviour, the constitutive equation for the stress and the heat flux can be expressed as

(3) $$\sigma_{ij}^{\epsilon }=D_{ijhk}^{\epsilon }\left(u_{h,k}^{\epsilon }-\alpha_{hk}^{\epsilon }\theta^{\epsilon }\right)\;\mathrm{and}\;q_{i}^{\epsilon }=-k_{ij}^{\epsilon }\theta_{,j}^{\epsilon }\;\mathrm{in}\;\Omega ,$$

where $u_{i}^{\epsilon }=u_{i}^{\epsilon }\left(x\right)$ and $\theta^{\epsilon }=\theta^{\epsilon }\left(x\right)$ are the unknown displacement and temperature variation field (respectively), $D_{ijhk}^{\epsilon }=D_{ijhk}^{\epsilon }\left(x\right)$ is the fourth-order elastic stiffness tensor, $\alpha_{hk}^{\epsilon }=\alpha_{hk}^{\epsilon }\left(x\right)$ is the second-order thermal expansion tensor and $k_{ij}^{\epsilon }=k_{ij}^{\epsilon }\left(x\right)$ is the second-order thermal conductivity tensor. If the constituent materials exhibit an isotropic behaviour, these latter properties can be expressed as

(4) $$D_{ijhk}^{\epsilon }=\mu^{\epsilon }\left(\delta_{ih}\delta_{jk}+\delta_{ik}\delta_{jh}\right)+\lambda^{\epsilon }\delta_{ij}\delta_{hk},\;\alpha_{hk}^{\epsilon }=\alpha^{\epsilon }\delta_{hk}\;\mathrm{and}\;k_{ij}^{\epsilon }=k^{\epsilon }\delta_{ij},$$

where $\lambda^{\epsilon }=\lambda^{\epsilon }\left(x\right)$ and $\mu^{\epsilon }=\mu^{\epsilon }\left(x\right)$ are Lame’s constant, $\alpha^{\epsilon }=\alpha^{\epsilon }\left(x\right)$ is the coefficient of thermal expansion and $k^{\epsilon }=k^{\epsilon }\left(x\right)$ is the coefficient of thermal conductivity. The heterogeneity of the metamaterial can be obtained by periodically combining different materials and holes. In this latter case, we will consider a void as a material with null properties.

Note that the material properties $D_{ijhk}^{\epsilon },\alpha_{hk}^{\epsilon }$ and $k_{ij}^{\epsilon }$ can be periodically heterogenous but do not depend on temperature, coherently with our fourth hypothesis.

On the top and bottom boundaries of the metamaterial, we prescribe zero normal traction and heat flux, i.e.,

(5) $$\sigma_{ij}^{\epsilon }n_{j}=0\;\mathrm{and}\;q_{i}^{\epsilon }n_{i}=0\;\mathrm{on}\;\Gamma_{+}^{\epsilon }\cup \Gamma_{-}^{\epsilon },$$

where $n_{i}$ is the outer unit normal vector on $\Gamma_{\pm }^{\epsilon }$. The boundary conditions on the lateral surfaces of the media, i.e., on $\partial \Omega \setminus (\Gamma_{+}^{\epsilon }\cup \Gamma_{-}^{\epsilon })$, will not affect the asymptotic homogenization procedure and will be therefore left unspecified. We refer to V(Ω) as a couple of fields $u^{\epsilon },\theta^{\epsilon }\in H^{1}\left(\Omega \right)$ satisfying all the prescribed boundary conditions.

The thermoelastic problem (2) can be reformulated as follows: find $\left(u^{\epsilon },\theta^{\epsilon }\right)\in V\left(\Omega \right)$ such that

(6) $$\int_{\Omega }\left(\sigma_{ij}^{\epsilon }v_{i,j}^{\epsilon }-F_{i}^{\epsilon }v_{i}^{\epsilon }+q_{i}\eta_{,i}^{\epsilon }+r^{\epsilon }\eta^{\epsilon }\right)\;dx=0\;\forall v_{i}^{\epsilon },\eta^{\epsilon }\in H_{0}^{1}\left(\Omega \right).$$

*Remark*—Any actual size of the unit cell can be considered, provided that it is small with respect to the whole system (first hypothesis) and that the constituent material behaviour can be described by classical continuum mechanics.

## 3. Asymptotic Homogenization

### 3.1. Scaling and Asymptotic Expansion

**Figure 2:**
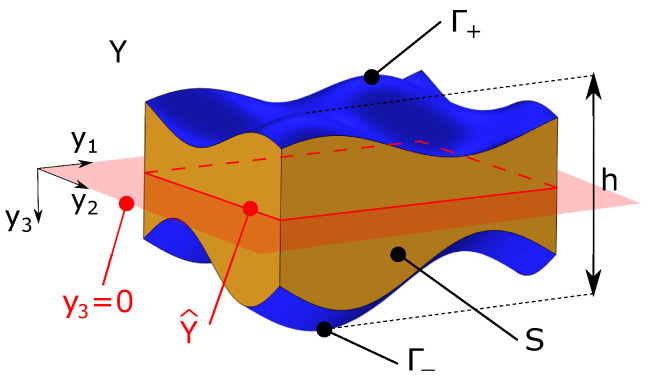
Re-scaled unit cell *Y* and the corresponding mid-surface $\hat{Y}$ (in red).

According to the two-scale homogenization technique, we introduce the fast variable y=x/ϵ which lives in the re-scaled unit cell $Y=Y^{\epsilon }/\epsilon$ of the periodic media, shown in Figure 2. The re-scaled unit cell mid-surface (in the red plane of the equation $y_{3}=0$) is denoted by $\hat{Y}$, the lateral surface (in orange) by *S*, and the top/bottom surface (in blue) by $\Gamma_{\pm }=\Gamma_{\pm }^{\epsilon }/\epsilon$. The latter have the equation $y_{3}=h_{\pm }\left(y_{1},y_{2}\right)=h_{\pm }^{\epsilon }\left(\epsilon y_{1},\epsilon y_{2}\right)/\epsilon$, while the maximum thickness of the unit cell is $h=h^{\epsilon }/\epsilon$.

The properties of the constituent materials, which vary within *Y*, are not scaled, i.e.,

(7) $$D_{ijhk}^{\epsilon }\left(x\right)=D_{ijhk}\left(\frac{x}{\epsilon }\right),\;\alpha_{hk}^{\epsilon }\left(x\right)=\alpha_{hk}\left(\frac{x}{\epsilon }\right)\;\mathrm{and}\;k_{ij}^{\epsilon }\left(x\right)=k_{ij}\left(\frac{x}{\epsilon }\right),$$

where $D_{ijhk}\left(y\right),\;\alpha_{hk}\left(y\right)$ and $k_{ij}\left(y\right)$ are defined on *Y* and are $\hat{Y}$-periodic with respect to $y_{1}$ and $y_{2}$ only.

As the thickness decreases as *ℓ* in the asymptotic limit ϵ→0, we need to scale body forces and heat production according to

(8) $$F_{\alpha }^{\epsilon }\left(x\right)=\epsilon F_{\alpha }\left(\hat{x},\frac{x}{\epsilon }\right),\;F_{3}^{\epsilon }\left(x\right)=\epsilon^{2}F_{3}\left(\hat{x},\frac{x}{\epsilon }\right)\;\mathrm{and}\;r^{\epsilon }\left(x\right)=\epsilon \;r\left(\hat{x},\frac{x}{\epsilon }\right),$$

with $F_{i}\left(\hat{x},y\right)$ and $r(\hat{x},y)$ being defined on $\hat{\Omega }\times Y$ and being $\hat{Y}$-periodic with respect to the variables $y_{1}$ and $y_{2}$.

With the scaling hypotheses given by Equations (7) and (8), the solution of problem (6) can be expanded in terms of ϵ. In particular, we assume for the displacement field and the stress tensor the following ansatzs

(9) $$\begin{array}{cc} \; & u_{i}^{\epsilon }\left(x\right)=u_{i}^{0}\left(\hat{x}\right)+\epsilon u_{i}^{1}\left(\hat{x},\frac{x}{\epsilon }\right)+\epsilon^{2}u_{i}^{2}\left(\hat{x},\frac{x}{\epsilon }\right)+o\left(\epsilon^{2}\right),\\ \; & \sigma_{ij}^{\epsilon }\left(x\right)=\sigma_{ij}^{0}\left(\hat{x},\frac{x}{\epsilon }\right)+\epsilon \sigma_{ij}^{1}\left(\hat{x},\frac{x}{\epsilon }\right)+\epsilon^{2}\sigma_{ij}^{2}\left(\hat{x},\frac{x}{\epsilon }\right)+o\left(\epsilon^{2}\right), \end{array}$$

where $u_{i}^{n}\left(\hat{x},y\right)$ and $\sigma_{ij}^{n}\left(\hat{x},y\right)$, for n∈N, are defined on $\hat{\Omega }\times Y$ and are $\hat{Y}$-periodic with respect to $y_{1}$ and $y_{2}$. Note that, the 0-th order displacement field $u^{0}$ is assumed to depend on the in-plane slow variable $\hat{x}$ only. Asymptotic expansions analogous to (9) hold for the temperature variation field $\theta^{\epsilon }\left(x\right)$ and the heat flux $q_{i}^{\epsilon }\left(x\right)$. For a variational consistency, we further assume a similar dependence on $\hat{x}$ and y of the test functions, i.e.,

(10) $$v_{i}^{\epsilon }\left(x\right)=v\left(\hat{x},\frac{x}{\epsilon }\right)\;\mathrm{and}\;\eta^{\epsilon }\left(x\right)=\eta \left(\hat{x},\frac{x}{\epsilon }\right),$$

with $v_{i}\left(\hat{x},y\right)$ and $\eta (\hat{x},y)$ being defined on $\hat{\Omega }\times Y$ and being $\hat{Y}-$ periodic with respect to $y_{1}$ and $y_{2}$. Note that, due to the chain rule, the derivatives of the function $f^{\epsilon }\left(x\right)=f\left(\hat{x},x/\epsilon \right)$ are computed as

(11) $$\begin{array}{c} f_{,\alpha }^{\epsilon }=f_{,\alpha }+\epsilon^{-1}f_{|\alpha }\;\mathrm{and}\;f_{,3}^{\epsilon }=\epsilon^{-1}f_{|3}. \end{array}$$

Using the relation between the coordinates $x_{3}=\epsilon y_{3}$, one has

(12) $$\int_{\Omega }\left(⋄\right)\;dx=\int_{\hat{\Omega }}\left(\int_{h_{-}^{\epsilon }\left(\hat{x}\right)}^{h_{+}^{\epsilon }\left(\hat{x}\right)}\left(⋄\right)\;dx_{3}\right)\;d\hat{x}=\epsilon \int_{\hat{\Omega }}\left(\int_{h_{-}\left(\hat{x}\right)}^{h_{+}\left(\hat{x}\right)}\left(⋄\right)\;dy_{3}\right)\;d\hat{x}.$$

We also introduce the averaging operator on the re-scaled unit cell

(13) $$\langle (⋄)\rangle =\frac{1}{\left|Y\right|}\int_{Y}\left(⋄\right)\;dy,$$

where |Y| is the volume of the re-scaled unit cell.

Using (11), (12) and (13) the weak form of the governing Equation (6) can be expressed as

(14) $$\int_{\hat{\Omega }}\langle \sigma_{ij}^{\epsilon }v_{i|j}+q_{i}^{\epsilon }\eta_{|i}+\epsilon \left(\sigma_{i\beta }^{\epsilon }v_{i,\beta }+q_{\alpha }^{\epsilon }\eta_{,\alpha }\right)+\epsilon^{2}\left(r\eta -F_{\alpha }v_{\alpha }\right)-\epsilon^{3}F_{3}v_{3}\rangle \;d\hat{x}=0,$$

for each possible $v_{i},\eta \in H_{\#}^{1}\left(\hat{\Omega }\times Y\right)$. Here, $H_{\#}^{1}\left(\hat{\Omega }\times Y\right)$ is the subspace of all the functions belonging to $H^{1}\left(\hat{\Omega }\times Y\right)$ that vanish on $\partial \hat{\Omega }\cup \Gamma_{+}\cup \Gamma_{-}$ and are $\hat{Y}-$ periodic on *S* with respect to $y_{1}$ and $y_{2}$. For functions depending on y only, we denote with $H_{\#}^{1}\left(Y\right)$ the analogous functional space.

Replacing the asymptotic expansions (9) into Equation (14), one obtains a sequence of problems $P(\epsilon^{n})$ for each order n∈N of the parameter ϵ. The first four problems read

(15) $$\begin{array}{cc} P(\epsilon^{0}):\; & \int_{\hat{\Omega }}\langle \sigma_{ij}^{0}v_{i|j}+q_{i}^{0}\eta_{|i}\rangle \;d\hat{x}=0,\\ P(\epsilon^{1}):\; & \int_{\hat{\Omega }}\langle \sigma_{ij}^{1}v_{i|j}+q_{i}^{1}\eta_{|i}+\sigma_{i\beta }^{0}v_{i,\beta }+q_{\alpha }^{0}\eta_{,\alpha }\rangle \;d\hat{x}=0,\\ P(\epsilon^{2}):\; & \int_{\hat{\Omega }}\langle \sigma_{ij}^{2}v_{i|j}+q_{i}^{2}\eta_{|i}+\sigma_{i\beta }^{1}v_{i,\beta }+q_{\alpha }^{1}\eta_{,\alpha }\rangle \;d\hat{x}=\int_{\hat{\Omega }}\langle F_{\alpha }v_{\alpha }-r\eta \rangle \;d\hat{x},\\ P(\epsilon^{3}):\; & \int_{\hat{\Omega }}\langle \sigma_{ij}^{3}v_{i|j}+q_{i}^{3}\eta_{|i}+\sigma_{i\beta }^{2}v_{i,\beta }+q_{\alpha }^{2}\eta_{,\alpha }\rangle \;d\hat{x}=\int_{\hat{\Omega }}\langle F_{3}v_{3}\rangle \;d\hat{x}, \end{array}$$

for each possible $v_{i},\eta \in H_{\#}^{1}\left(\hat{\Omega }\times Y\right)$. Note that, making use of Equations (3) and (11), one can compute the expansion terms of stress and heat flux as

(16) $$\sigma_{ij}^{n}=D_{ijh\delta }u_{h,\delta }^{n}+D_{ijhk}\left(u_{h|k}^{n+1}-\alpha_{hk}\theta^{n}\right)\;\mathrm{and}\;q_{i}^{n}=-\left(k_{i\beta }\theta_{,\beta }^{n}+k_{ij}\theta_{|j}^{n+1}\right),$$

for n∈N.

### 3.2. Thermal-Conductivity Problem

As can be observed from the governing equations, the steady-state thermoelastic problem is weakly coupled, i.e., the thermal equilibrium configuration does not depend on the solution of the mechanical problem. Therefore, we can solve first the thermal conductivity homogenization problem by assuming for all problems (15) that $v_{i}=0$.

#### 3.2.1. Problem $P(\epsilon^{0})$

Choosing $\eta \left(\hat{x},y\right)=\varphi \left(\hat{x}\right)\psi \left(y\right)$ and recalling that $q_{i}^{0}=-\left(k_{i\beta }\theta_{,\beta }^{0}+k_{ij}\theta_{|j}^{1}\right)$, one obtains

(17) $$\langle \left(k_{i\beta }\theta_{,\beta }^{0}+k_{ij}\theta_{|j}^{1}\right)\psi_{|i}\rangle =0\;\forall \psi \in H_{\#}^{1}\left(Y\right),$$

which implies, by linearity, that the solution is given by

(18) $$\theta^{1}\left(\hat{x},y\right)=\theta^{*}\left(\hat{x}\right)+\theta_{,\beta }^{0}\left(\hat{x}\right)\Theta^{\beta }\left(y\right)\;\mathrm{in}\;\hat{\Omega }\times Y.$$

In Equation (18), $\theta^{*}\left(\hat{x}\right)$ is the homogenized temperature variation field dependent only on the macroscopic in-plane variable, while $\Theta^{\beta }\left(y\right)$ is the solution of the *thermal conductivity cell problem*

(19) $$\left\{\begin{array}{cc} {\left[k_{ij}\left(\delta_{j\beta }+\Theta_{|j}^{\beta }\right)\right]}_{|i}=0 & \mathrm{in}\;Y,\\ \Theta^{\beta }\;\mathrm{periodic} & \mathrm{on}\;S,\\ \left[k_{ij}\left(\delta_{j\beta }+\Theta_{|j}^{\beta }\right)\right]n_{i}\;\mathrm{anti-periodic} & \mathrm{on}\;S,\\ \left[k_{ij}\left(\delta_{j\beta }+\Theta_{|j}^{\beta }\right)\right]n_{i}=0 & \mathrm{on}\;\Gamma_{+}\cup \Gamma_{-}. \end{array}\right.$$

#### 3.2.2. Problem $P(\epsilon^{1})$

Selecting now $\eta \left(\hat{x},y\right)=\varphi \left(\hat{x}\right)$, and making use of (18), problem $P(\epsilon^{1})$ reduces to

(20) $$\int_{\hat{\Omega }}k_{\alpha \beta }^{*}\theta_{,\beta }^{0}\varphi_{,\alpha }\;d\hat{x}=0\;\forall \varphi \in H_{0}^{1}\left(\hat{\Omega }\right),$$

where we have introduced the *homogenized in-plane thermal conductivity tensor* of the metaplate

(21) $$k_{\alpha \beta }^{*}=\frac{1}{\left|Y\right|}\int_{Y}k_{\alpha j}\left(\delta_{j\beta }+\Theta_{|j}^{\beta }\right)\;dy.$$

The tensor $k_{\alpha \beta }^{*}$ can be proved to be symmetric and positive definite [10]. Thus, from Equation (20) one deduces that $\theta^{0}\left(\hat{x}\right)=0$ and therefore, from (18), that $\theta^{1}\left(\hat{x},y\right)=\theta^{*}\left(\hat{x}\right)$.

Considering now another test function $\eta \left(\hat{x},y\right)=\varphi \left(\hat{x}\right)\psi \left(y\right)$, problem $P(\epsilon^{1})$ allows us to obtain

(22) $$\langle \left(k_{i\beta }\theta_{,\beta }^{*}+k_{ij}\theta_{|j}^{2}\right)\psi_{|i}\rangle =0\;\forall \psi \in H_{\#}^{1}\left(Y\right),$$

which means, similarly to the previous problem, that

(23) $$\theta^{2}\left(\hat{x},y\right)=\theta_{,\beta }^{*}\left(\hat{x}\right)\Theta^{\beta }\left(y\right)\;\mathrm{in}\;\hat{\Omega }\times Y.$$

#### 3.2.3. Problem $P(\epsilon^{2})$

Selecting $\eta \left(\hat{x},y\right)=\varphi \left(\hat{x}\right)$ for the problem at the second order, we obtain

(24) $$\int_{\hat{\Omega }}\left(-k_{\alpha \beta }^{*}\theta_{,\beta }^{*}\varphi_{,\alpha }+\langle r\rangle \varphi \right)\;d\hat{x}=0\;\forall \varphi \in H_{0}^{1}\left(\hat{\Omega }\right),$$

which can be recognized as the weak form of the *homogenized thermal conductivity problem*

(25) $${\left(-k_{\alpha \beta }^{*}\theta_{,\beta }^{*}\right)}_{,\alpha }=r^{*}\;\mathrm{in}\;\hat{\Omega }.$$

In Equation (25), $r^{*}=\langle r\rangle$ is the effective heat production in the homogenized metaplate.

### 3.3. Mechanical Problem

After the homogenization of the thermal conductivity problem, we can now focus on the mechanical one by considering the sequence of problems (15) with η=0.

#### 3.3.1. Problem $P(\epsilon^{0})$

Starting from the problem at order zero, assuming $v_{i}\left(\hat{x},y\right)=\varphi \left(\hat{x}\right)\psi_{i}\left(y\right)$, one obtains

(26) $$\langle \left(D_{ijh\delta }u_{h,\delta }^{0}+D_{ijhk}u_{h|k}^{1}\right)\psi_{i|j}\rangle =0\;\forall \psi_{i}\in H_{\#}^{1}\left(Y\right).$$

The temperature variation field, which starts its asymptotic expansion at the first order since $\theta^{0}=0$ (see Section 3.2.2), is not involved in the formulation of the problem (26). Therefore, the solution is exactly coincident with the one obtained in [26] and reads

(27) $$\begin{array}{cc} u_{\gamma }^{1}\left(\hat{x},y\right) & =U_{\gamma }^{1}\left(\hat{x}\right)+u_{\alpha ,\beta }^{0}\left(\hat{x}\right)\chi_{h}^{\alpha \beta }\left(y\right)-y_{3}u_{3,\gamma }^{0}\\ u_{3}^{1}\left(\hat{x},y\right) & =U_{3}^{1}\left(\hat{x}\right)+u_{\alpha ,\beta }^{0}\left(\hat{x}\right)\chi_{3}^{\alpha \beta }\left(y\right) \end{array}\;\mathrm{in}\;\hat{\Omega }\times Y,$$

where $U_{h}^{1}\left(\hat{x}\right)$ is still undermined and $\chi_{h}^{\alpha \beta }\left(y\right)$, for α,β∈{1,2}, is the solution of the *membrane cell problem*

(28) $$\left\{\begin{array}{cc} {\left[D_{ijhk}\left(\delta_{h\alpha }\delta_{k\beta }+\chi_{h|k}^{\alpha \beta }\right)\right]}_{|j}=0 & \mathrm{in}\;Y,\\ \chi_{h}^{\alpha \beta }\;\mathrm{periodic} & \mathrm{on}\;S,\\ \left[D_{ijhk}\left(\delta_{h\alpha }\delta_{k\beta }+\chi_{h|k}^{\alpha \beta }\right)\right]n_{j}\;\mathrm{anti-periodic} & \mathrm{on}\;S,\\ \left[D_{ijhk}\left(\delta_{h\alpha }\delta_{k\beta }+\chi_{h|k}^{\alpha \beta }\right)\right]n_{j}=0 & \mathrm{on}\;\Gamma_{+}\cup \Gamma_{-}. \end{array}\right.$$

The field $\chi_{h}^{\alpha \beta }$ represents the h-component of the displacement field when a uniform in-plane eigenstrain $e_{\alpha }⊙e_{\beta }$ is applied within the unit cell subjected to periodicity conditions on *S* and zero-traction on $\Gamma_{+}\cup \Gamma_{-}$. The solution of problem (28) is defined up to an arbitrary constant, which represents a rigid body motion of the unit cell. Only three membrane cell problems need to be solved since it is possible to show that $\chi_{h}^{\alpha \beta }=\chi_{h}^{\beta \alpha }$.

#### 3.3.2. Problem $P(\epsilon^{1})$

When considering the next order, following the same developments of [26], it is possible to show that $u_{\gamma }^{0}=0$ and thus Equation (27) reduces to

(29) $$u_{\gamma }^{1}\left(\hat{x},y\right)=u_{\gamma }^{*}\left(\hat{x}\right)-y_{3}w_{,\gamma }^{*}\left(\hat{x}\right)\;\mathrm{and}\;u_{3}^{1}\left(\hat{x},y\right)=U_{3}^{1}\left(\hat{x}\right)\;\mathrm{in}\;\hat{\Omega }\times Y,$$

where we have introduced the homogenized in-plane and out-of-plane displacements $u_{\gamma }^{*}=U_{\gamma }^{1}$ and $w^{*}=u_{3}^{0}$, respectively.

Assuming $v_{i}\left(\hat{x},y\right)=\varphi \left(\hat{x}\right)\psi_{i}\left(y\right)$, problem $P(\epsilon^{1})$ gives

(30) $$\langle \left[D_{ijh\delta }u_{h,\delta }^{*}-y_{3}D_{ij\gamma \delta }w_{,\gamma \delta }^{*}+D_{ijhk}\left(u_{h|k}^{2}-\alpha_{hk}\theta^{*}\right)\right]\psi_{i|j}\rangle =0\;\forall \psi_{i}\in H_{0}^{1}\left(\hat{\Omega }\right),$$

which now involves the effective temperature variation field $\theta^{*}\left(\hat{x}\right)$ solution of the homogenized thermal conductivity problem (25). The solution of (30) can be expressed by linearity as

(31) $$\begin{array}{cc} \; & u_{\gamma }^{2}\left(\hat{x},y\right)=u_{\alpha ,\beta }^{*}\left(\hat{x}\right)\chi_{\gamma }^{\alpha \beta }-w_{,\alpha \beta }^{*}\left(\hat{x}\right)\xi_{\gamma }^{\alpha \beta }\left(y\right)-\theta^{*}\left(\hat{x}\right)\zeta_{\gamma }\left(y\right)-y_{3}U_{3,\gamma }^{1}\left(\hat{x}\right)\\ \; & u_{3}^{2}\left(\hat{x},y\right)=u_{\alpha ,\beta }^{*}\left(\hat{x}\right)\chi_{3}^{\alpha \beta }-w_{,\alpha \beta }^{*}\left(\hat{x}\right)\xi_{3}^{\alpha \beta }\left(y\right)-\theta^{*}\left(\hat{x}\right)\zeta_{3}\left(y\right) \end{array}\;\mathrm{in}\;\hat{\Omega }\times Y,$$

where $\xi_{h}^{\alpha \beta }\left(y\right)$ solves, for α,β∈{1,2}, the *flexural cell problem*

(32) $$\left\{\begin{array}{cc} {\left[D_{ijhk}\left(y_{3}\delta_{h\alpha }\delta_{k\beta }+\xi_{h|k}^{\alpha \beta }\right)\right]}_{|j}=0 & \mathrm{in}\;Y\\ \xi_{h}^{\alpha \beta }\;\mathrm{periodic} & \mathrm{on}\;S\\ \left[D_{ijhk}\left(y_{3}\delta_{h\alpha }\delta_{k\beta }+\xi_{h|k}^{\alpha \beta }\right)\right]n_{j}\;\mathrm{anti-periodic} & \mathrm{on}\;S\\ \left[D_{ijhk}\left(y_{3}\delta_{h\alpha }\delta_{k\beta }+\xi_{h|k}^{\alpha \beta }\right)\right]n_{j}=0 & \mathrm{on}\;\Gamma_{+}\cup \Gamma_{-} \end{array}\right.$$

while $\zeta_{h}\left(y\right)$ solves the *thermoelastic cell problem*

(33) $$\left\{\begin{array}{cc} {\left[D_{ijhk}\left(\alpha_{hk}+\zeta_{h|k}\right)\right]}_{|j}=0 & \mathrm{in}\;Y\\ \zeta_{h}\;\mathrm{periodic} & \mathrm{on}\;S\\ \left[D_{ijhk}\left(\alpha_{hk}+\zeta_{h|k}\right)\right]n_{j}\;\mathrm{anti-periodic} & \mathrm{on}\;S\\ \left[D_{ijhk}\left(\alpha_{hk}+\zeta_{h|k}\right)\right]n_{j}=0 & \mathrm{on}\;\Gamma_{+}\cup \Gamma_{-} \end{array}\right.$$

The field $\xi_{h}^{\alpha \beta }\left(y\right)$ represents the h-component of the displacement field of the unit cell, subject to periodic boundary conditions on *S* and zero traction on $\Gamma_{+}\cup \Gamma_{-}$, when an eigenstrain $y_{3}e_{\alpha }⊙e_{\beta }$ linearly varying with $y_{3}$ is applied within *Y*. The field $\zeta_{h}\left(y\right)$ is the h-component of the displacement due to a uniform unit temperature variation in the re-scaled cell by considering analogous boundary conditions. Both the solution of cell problems (32) and (33) are defined up to a constant term, which represents a rigid body motion of the unit cell. Note that, due to the fact that $\xi_{h}^{\alpha \beta }=\xi_{h}^{\beta \alpha }$, only three flexural cell problems must be solved numerically.

With the definition of the stress localization tensors

(34) $$\begin{array}{cc} \; & a_{ij\gamma \delta }^{*}\left(y\right)=D_{ijhk}\left(y\right)\left(\delta_{h\gamma }\delta_{k\delta }+\chi_{h|k}^{\gamma \delta }\left(y\right)\right)\\ \; & b_{ij\gamma \delta }^{*}\left(y\right)=D_{ijhk}\left(y\right)\left(y_{3}\delta_{h\gamma }\delta_{k\delta }+\xi_{h|k}^{\gamma \delta }\left(y\right)\right)\\ \; & t_{ij}^{*}\left(y\right)=D_{ijhk}\left(y\right)\left(\alpha_{hk}\left(y\right)+\zeta_{h|k}\left(y\right)\right) \end{array}\;\mathrm{in}\;\hat{\Omega }\times Y,$$

the first-order stress tensor can be expressed, making use of (31), as

(35) $$\sigma_{ij}^{1}\left(\hat{x},y\right)=a_{ij\gamma \delta }^{*}\left(y\right)u_{\gamma ,\delta }^{*}\left(\hat{x}\right)-b_{ij\gamma \delta }^{*}\left(y\right)w_{,\gamma \delta }^{*}\left(\hat{x}\right)-t_{ij}^{*}\left(y\right)\theta^{*}\left(\hat{x}\right)\;\mathrm{in}\;\hat{\Omega }\times Y.$$

#### 3.3.3. Problems $P(\epsilon^{2})$ and $P(\epsilon^{3})$

Following the same steps as in [26], solving the problems $P(\epsilon^{2})$ and $P(\epsilon^{3})$, one obtains the *effective equilibrium equations of the homogenized plate*, which read

(36) $$\left\{\begin{array}{c} N_{\alpha \beta ,\beta }^{*}+p_{\alpha }^{*}=0\\ M_{\alpha \beta ,\alpha \beta }^{*}+p_{3}^{*}=0 \end{array}\right.\;\mathrm{in}\;\hat{\Omega }.$$

Equation (36) can be recognized as the governing equation of a Kirchhoff–Love plate, having defined the homogenized plate membrane forces and moments as

(37) $$N_{\alpha \beta }^{*}\left(\hat{x}\right)=h^{*}\langle \sigma_{\alpha \beta }^{1}\left(\hat{x},y\right)\rangle \;M_{\alpha \beta }^{*}\left(\hat{x}\right)=h^{*}\langle y_{3}\sigma_{\alpha \beta }^{1}\left(\hat{x},y\right)\rangle \;\mathrm{in}\;\hat{\Omega },$$

and the membrane and out-of-plane homogenized load as

(38) $$p_{\alpha }^{*}=h^{*}\langle F_{\alpha }\rangle \;\mathrm{and}\;p_{3}^{*}=h^{*}\left[\langle F_{3}\rangle +\langle y_{3}F_{\alpha ,\alpha }\rangle \right].$$

In Equations (37) and (38), $h^{*}$ is the homogenized plate thickness that is defined as the ratio between the volume of the re-scaled unit cell *Y* and the area of its mid-surface $\hat{Y}$.

### 3.4. Effective Thermoelastic Properties

The effective stiffnesses of the homogenized metamaterial plate can be retrieved from the constitutive law of the membrane forces $N_{\alpha \beta }^{*}$ and plate moments $M_{\alpha \beta }^{*}$. Replacing (35) into the definitions (37), one finally has

(39) $$\begin{array}{c} N_{\alpha \beta }^{*}\left(\hat{x}\right)=A_{\alpha \beta \gamma \delta }^{*}u_{\gamma ,\delta }^{*}\left(\hat{x}\right)-C_{\alpha \beta \gamma \delta }^{*}w_{,\gamma \delta }^{*}\left(\hat{x}\right)-E_{\alpha \beta }^{*}\theta^{*}\left(\hat{x}\right)\\ M_{\alpha \beta }^{*}\left(\hat{x}\right)=C_{\gamma \delta \alpha \beta }^{*}u_{\gamma ,\delta }^{*}\left(\hat{x}\right)-B_{\alpha \beta \gamma \delta }^{*}w_{,\gamma \delta }^{*}\left(\hat{x}\right)-F_{\alpha \beta }^{*}\theta^{*}\left(\hat{x}\right) \end{array}\;\mathrm{in}\;\hat{\Omega },$$

where the following homogenized stiffnesses and generalized plate thermal stresses have been defined:

(40) $$\begin{array}{cc} \; & A_{\alpha \beta \gamma \delta }^{*}=h^{*}\langle a_{\alpha \beta \gamma \delta }^{*}\left(y\right)\rangle \;\mathrm{homog}.\;\mathrm{membrane}\;\mathrm{stiffness},\\ \; & B_{\alpha \beta \gamma \delta }^{*}=h^{*}\langle y_{3}b_{\alpha \beta \gamma \delta }^{*}\left(y\right)\rangle \;\mathrm{homog}.\;\mathrm{bending}\;\mathrm{stiffness},\\ \; & C_{\alpha \beta \gamma \delta }^{*}=h^{*}\langle b_{\alpha \beta \gamma \delta }^{*}\left(y\right)\rangle =h^{*}\langle y_{3}a_{\gamma \delta \alpha \beta }^{*}\left(y\right)\rangle \;\mathrm{homog}.\;\mathrm{coupling}\;\mathrm{stiffness},\\ \; & E_{\alpha \beta }^{*}=h^{*}\langle t_{\alpha \beta }^{*}\left(y\right)\rangle \;\mathrm{homog}.\;\mathrm{thermal}\;\mathrm{membrane}\;\mathrm{forces},\\ \; & F_{\alpha \beta }^{*}=h^{*}\langle y_{3}t_{\alpha \beta }^{*}\left(y\right)\rangle \;\mathrm{homog}.\;\mathrm{thermal}\;\mathrm{plate}\;\mathrm{moments}. \end{array}$$

The fourth-order tensors $A_{\alpha \beta \gamma \delta }^{*}$ and $B_{\alpha \beta \gamma \delta }^{*}$ have minor and major symmetries; thus, recalling that α,β,γ,δ∈{1,2}, they are characterized by six independent components each. The tensor $C_{\alpha \beta \gamma \delta }^{*}$ has minor symmetries while, in general, it does not have major symmetries, i.e., $C_{\alpha \beta \gamma \delta }^{*}\neq C_{\gamma \delta \alpha \beta }^{*}$ (9 independent components). The second-order tensors $E_{\alpha \beta }^{*}$ and $F_{\alpha \beta }^{*}$ are symmetric and described by three independent components.

Equation (39) describes the thermoelastic behaviour of the homogenized plate: it links the generalized plate stresses $N_{\alpha \beta }^{*}$ and $M_{\alpha \beta }^{*}$ to the in-plane $u_{\gamma }^{*}$ and out-of-plane $w^{*}$ displacement components of the homogenized plate mid-surface and to the homogenized temperature variation $\theta^{*}$.

The homogenized thermal expansion tensor $\alpha_{\gamma \delta }^{*}$ and the homogenized thermal-induced curvature $\kappa_{\gamma \delta }^{*}$ can be implicitly defined by the relations

(41) $$\begin{array}{cc} N_{\alpha \beta }^{*}\left(\hat{x}\right) & =A_{\alpha \beta \gamma \delta }^{*}\left(u_{\gamma ,\delta }^{*}\left(\hat{x}\right)-\alpha_{\gamma \delta }^{*}\theta^{*}\left(\hat{x}\right)\right)-C_{\alpha \beta \gamma \delta }^{*}\left(w_{,\gamma \delta }^{*}\left(\hat{x}\right)+\kappa_{\gamma \delta }^{*}\theta^{*}\left(\hat{x}\right)\right)\\ M_{\alpha \beta }^{*}\left(\hat{x}\right) & =C_{\gamma \delta \alpha \beta }^{*}\left(u_{\gamma ,\delta }^{*}\left(\hat{x}\right)-\alpha_{\gamma \delta }^{*}\theta^{*}\left(\hat{x}\right)\right)-B_{\alpha \beta \gamma \delta }^{*}\left(w_{,\gamma \delta }^{*}\left(\hat{x}\right)+\kappa_{\gamma \delta }^{*}\theta^{*}\left(\hat{x}\right)\right) \end{array}\;\mathrm{in}\;\hat{\Omega }.$$

A direct comparison of Equations (39) and (41) allows the identification of $\alpha_{\gamma \delta }^{*}$ and $\kappa_{\gamma \delta }^{*}$ as the solutions of the linear system

(42) $$\left\{\begin{array}{c} A_{\alpha \beta \gamma \delta }^{*}\alpha_{\gamma \delta }^{*}+C_{\alpha \beta \gamma \delta }^{*}\kappa_{\gamma \delta }^{*}=E_{\alpha \beta }^{*},\\ C_{\gamma \delta \alpha \beta }^{*}\alpha_{\gamma \delta }^{*}+B_{\alpha \beta \gamma \delta }^{*}\kappa_{\gamma \delta }^{*}=F_{\alpha \beta }^{*}. \end{array}\right.$$

## 4. General Remarks

### 4.1. Back-Scaling of the Solution

When considering a real metaplate characterized by a finite value of ϵ, $\epsilon_{0}>0$, the homogenized solution needs to be scaled back to the real problem. This can be achieved by looking at the leading terms in the asymptotic expansion (9). Denoting by ${\left(⋄\right)}^{h}$ the reconstruction of the field (⋄) through the homogenization procedure, one has for the temperature variation and displacement fields

(43) $$\theta^{h}\left(x\right)=\epsilon_{0}\theta^{*}\left(\hat{x}\right),\;u_{\gamma }^{h}\left(x\right)=\epsilon_{0}u_{\gamma }^{*}\left(\hat{x}\right)-x_{3}w_{,\gamma }^{*}\left(\hat{x}\right)\;\mathrm{and}\;u_{3}^{h}\left(x\right)=w^{*}\left(\hat{x}\right)\;\mathrm{in}\;\Omega .$$

Note that the periodicity, and hence $\epsilon_{0}$, does not affect the out-of-plane displacement $u_{3}^{h}$. The back-scaling of the local stress tensor, at each point $\hat{x}\in \hat{\Omega }$ within the unit cell *Y*, reads

(44) $$\sigma_{ij}^{h}\left(x,y\right)=\epsilon_{0}\left(a_{ij\gamma \delta }^{*}\left(y\right)u_{\gamma ,\delta }^{*}\left(\hat{x}\right)-b_{ij\gamma \delta }^{*}\left(y\right)w_{,\gamma \delta }^{*}\left(\hat{x}\right)-t_{ij}^{*}\left(y\right)\theta^{*}\left(\hat{x}\right)\right)\;\mathrm{in}\;\hat{\Omega }\times Y.$$

Similar considerations hold for the back-scaling of the effective generalized plate stresses, i.e., $N_{\alpha \beta }^{h}=\epsilon_{0}^{2}N_{\alpha \beta }^{*}$ and $M_{\alpha \beta }^{h}=\epsilon_{0}^{3}M_{\alpha \beta }^{*}$, and for the plate effective thermal properties, e.g., $\alpha_{\gamma \delta }^{h}=\alpha_{\gamma \delta }^{*}$ and $\kappa_{\gamma \delta }^{h}=\epsilon_{0}^{-1}k_{\gamma \delta }^{*}$.

### 4.2. Change of the Reference Mid-Plane

The whole homogenization procedure, and thus all the homogenized properties of the metamaterial, is referred to the chosen mid-surface $\hat{\Omega }$ in the plane $x_{3}=0$. If one wants to refer to a different plane, e.g., the one of equation $x_{3}=e$, with *e* being the eccentricity, a simple transformation holds for all the effective properties and loadings.

Denoting by $\tilde{\left(⋄\right)}$ the quantity (⋄) evaluated with respect to the shifted mid-surface ${\tilde{x}}_{3}=x_{3}-e=0$, it can be easily shown from (28), (32) and (33) that

(45) $${\tilde{\chi }}_{h}^{\alpha \beta }=\chi_{h}^{\alpha \beta },\;{\tilde{\xi }}_{h}^{\alpha \beta }=\xi_{h}^{\alpha \beta }-e\chi_{h}^{\alpha \beta }\;\mathrm{and}\;{\tilde{\zeta }}_{h}=\zeta_{h}\;\mathrm{in}\;Y.$$

From Equation (34), one has

(46) $${\tilde{a}}_{\alpha \beta \gamma \delta }^{*}=a_{\alpha \beta \gamma \delta }^{*}\;{\tilde{b}}_{\alpha \beta \gamma \delta }^{*}=b_{\alpha \beta \gamma \delta }^{*}-ea_{\alpha \beta \gamma \delta }^{*}\;\mathrm{and}\;{\tilde{t}}_{\alpha \beta \gamma \delta }^{*}=t_{\alpha \beta \gamma \delta }^{*}\;\mathrm{in}\;Y.$$

The homogenized properties (40) transform accordingly to

(47) $$\begin{array}{cc} \; & {\tilde{A}}_{\alpha \beta \gamma \delta }^{*}=A_{\alpha \beta \gamma \delta }^{*},\\ \; & {\tilde{B}}_{\alpha \beta \gamma \delta }^{*}=B_{\alpha \beta \gamma \delta }^{*}-e\left(C_{\alpha \beta \gamma \delta }^{*}+C_{\gamma \delta \alpha \beta }^{*}\right)+e^{2}A_{\alpha \beta \gamma \delta }^{*},\\ \; & {\tilde{C}}_{\alpha \beta \gamma \delta }^{*}=C_{\alpha \beta \gamma \delta }^{*}-eA_{\alpha \beta \gamma \delta }^{*},\\ \; & {\tilde{E}}_{\alpha \beta }^{*}=E_{\alpha \beta }^{*},\\ \; & {\tilde{F}}_{\alpha \beta }^{*}=F_{\alpha \beta }^{*}-eE_{\alpha \beta }^{*}. \end{array}$$

Exploiting Equation (47), the effective thermal expansion and curvature, solutions of (42), now read

(48) $${\tilde{\alpha }}_{\gamma \delta }^{*}=\alpha_{\gamma \delta }^{*}+e\kappa_{\gamma \delta }^{*}\;\mathrm{and}\;{\tilde{\kappa }}_{\gamma \delta }^{*}=\kappa_{\gamma \delta }^{*}.$$

From Equation (27), the displacements of the mid-surface change accordingly to

(49) $${\tilde{u}}_{\gamma }^{*}={\tilde{u}}_{\gamma }^{*}-ew_{,\gamma }^{*}\;\mathrm{and}\;{\tilde{w}}^{*}=w^{*},$$

and therefore, from (39), the plate internal actions become

(50) $${\tilde{N}}_{\alpha \beta }^{*}=N_{\alpha \beta }^{*}\;\mathrm{and}\;{\tilde{M}}_{\alpha \beta }^{*}=M_{\alpha \beta }^{*}-eN_{\alpha \beta }^{*}.$$

Finally, from Equation (38), the homogenized loads transform as

(51) $${\tilde{p}}_{\alpha }^{*}=p_{\alpha }^{*}\;\mathrm{and}\;{\tilde{p}}_{3}^{*}=p_{3}^{*}-ep_{\alpha ,\alpha }^{*}$$

### 4.3. The Case of Symmetric Metaplates

When $x_{3}=0$ is a plane of symmetry of the metamaterial plate, several simplifications arise. From Equations (28), (32) and (33) it is clear that $\xi_{h}^{\alpha \beta }\left(y\right)$ and $\zeta_{h}\left(y\right)$ are even functions of $y_{3}$, while $\xi_{h}^{\alpha \beta }\left(y\right)$ is odd in $y_{3}$. From (34), one has that $a_{\alpha \beta \gamma \delta }^{*}\left(y\right)$ and $t_{\alpha \beta \gamma \delta }^{*}\left(y\right)$ are even in $y_{3}$, while $b_{\alpha \beta \gamma \delta }^{*}\left(y\right)$ is odd with respect to $y_{3}$. Therefore, from (40), one can conclude that

(52) $$C_{\alpha \beta \gamma \delta }^{*}=C_{\gamma \delta \alpha \beta }^{*}=F_{\alpha \beta }^{*}=0,$$

which implies, by the second equation of (42), that

(53) $$\kappa_{\gamma \delta }^{*}=0.$$

As expected, for a metaplate with transverse symmetry, the membrane–bending coupling vanishes, as well as the effective thermal-induced bending.

### 4.4. The Case of Homogeneous Thermal Expansion Coefficient

Let us consider the case of a single homogeneous constituent material with periodically varying thickness and voids, or the case of periodically heterogeneous materials with very similar thermal expansion coefficients (e.g., concrete and steel). In such a case, denoting with $Y_{m}$ the portion of *Y* occupied by the material, the thermal expansion tensor has the expression

(54) $$\alpha_{hk}\left(y\right)=\left\{\begin{array}{cc} \alpha_{hk} & \mathrm{in}\;Y_{m},\\ 0 & \mathrm{in}\;Y\setminus Y_{m}. \end{array}\right.$$

Thus, the thermoelastic cell problem (33) admits the following close-form solution

(55) $$\zeta_{h}\left(y\right)=\alpha_{\gamma \delta }\chi_{h}^{\gamma \delta }\left(y\right)-y_{3}\left(2\alpha_{13}\delta_{1h}+2\alpha_{23}\delta_{2h}+\alpha_{33}\delta_{3h}\right)\;\mathrm{in}\;Y_{m}.$$

Replacing (55) into (34), one has

(56) $$t_{ij}^{*}\left(y\right)=a_{ij\gamma \delta }^{*}\left(y\right)\alpha_{\gamma \delta }\;\mathrm{in}\;Y_{m},$$

which means, from Equation (40), that the thermal membrane forces and the thermal plate moments read

(57) $$E_{\alpha \beta }^{*}=A_{\alpha \beta \gamma \delta }^{*}\alpha_{\gamma \delta }\;\mathrm{and}\;F_{\alpha \beta }^{*}=C_{\gamma \delta \alpha \beta }^{*}\alpha_{\gamma \delta }.$$

Making use of (57), the solution of the linear system (42) is

(58) $$\alpha_{\gamma \delta }^{*}=\alpha_{\gamma \delta }\;\mathrm{and}\;\kappa_{\gamma \delta }^{*}=0,$$

which means that the homogenized plate does not exhibit any thermal bending and has the same thermal expansion tensor as the constituent material. This could be considered as a proof of the well-known property that at least two materials with different thermal expansion coefficients are required in order to modify the effective thermal properties of the metamaterial.

## 5. Numerical Examples

The numerical examples discussed in this section are referred to MEMS scale applications, even if the homogenization technique can be employed at higher scales, and are carried out with the commercial finite element software COMSOL Multiphysics 6.1^®^. Properties of the employed materials at 20 °C are listed in Table 1. For silicon, these values characterize the polysilicon obtained by epitaxial growth for MEMS applications [8], while for nickel we assume the typical values of the bulk material. For real applications in MEMS devices, nickel properties could depend on the fabrication techniques and further characterization would be required.

### 5.1. Parametric Studies

As a first example, we show how the proposed asymptotic homogenization procedure can be effectively employed to perform parametric studies to identify the effective properties of a periodic thermoelastic plate.

**Figure 3:**
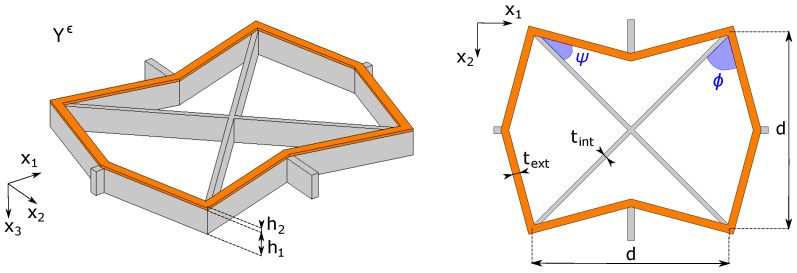
Unit cell employed for parametric studies: three-dimensional view (**left**) and top two-dimensional view (**right**).

We consider the unit cell shown in Figure 3, similar to the recently proposed one in [9], which is composed of two layers in the $x_{3}$-direction. The first one, in grey, constitutes the base structure of the cell; it has a height $h_{1}$ and it is made of silicon. The in-plane geometry of the first layer is characterized by two diagonal elements at ±45° of thickness $t_{\mathrm{int}}$ and length $d\sqrt{2}$, an external frame of thickness $t_{\mathrm{ext}}$, being in general non-convex, and by four lateral connectors that link the cell with the adjacent ones. The inclinations of the external frame are measured with respect to the diagonals and are indicated with ϕ (for the left and right elements) and ψ (for the top and bottom ones). The second layer, of height $h_{2}$, consists of a partial metallization made of nickel on the top of the external frame of the silicon base structure, as shown in Figure 3 in orange.

For the sake of simplicity, we fix the values $h_{1}=10h_{2}=10\;$µm, d=90µm and $t_{\mathrm{ext}}=t_{\mathrm{int}}=2\;$µm to perform the parametric studies by varying the inclination angles ϕ,ψ∈[10°,90°] only. For all calculations, we choose as the reference mid-surface the mid-plane of the silicon layer.

For each couple of values of (ϕ,ψ) we need to compute numerically the solutions $\chi_{h}^{11}$, $\chi_{h}^{22}$ and $\chi_{h}^{12}$ of the membrane cell problems (28), the solutions $\xi_{h}^{11}$, $\xi_{h}^{22}$ and $\xi_{h}^{12}$ of the flexural cell problems (32) and the solution $\zeta_{h}$ of the thermoelastic cell problem (33).

**Figure 4:**
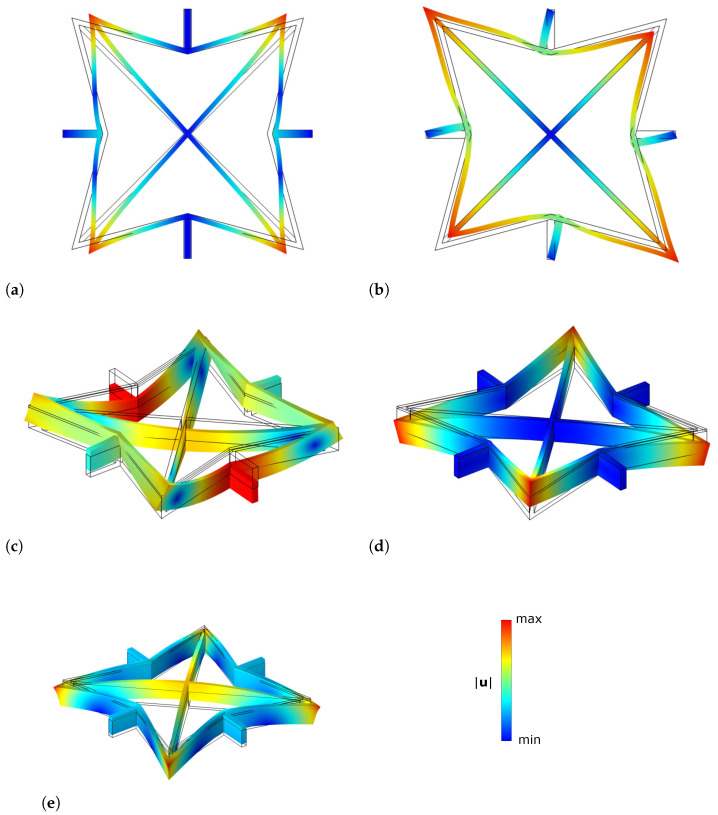
Contour of displacement magnitude of the solution of cell problems: (**a**) $\chi_{h}^{11}$, (**b**) $\chi_{h}^{12}$, (**c**) $\xi_{h}^{11}$, (**d**) $\xi_{h}^{12}$ and (**e**) $\zeta_{h}$ in the case $h_{1}=10h_{2}=10\;$µm, d=90µm, $t_{\mathrm{ext}}=t_{\mathrm{int}}=2\;$µm and ϕ=ψ=30°.

For example, in the case ϕ=ψ=30°, Figure 4a and Figure 4b show the contour of the displacement magnitude for the membrane cell problem $\chi_{h}^{11}$ and $\chi_{h}^{12}$ on the deformed shapes. Similarly, solutions for the flexural cell problems $\xi_{h}^{11}$ and $\xi_{h}^{12}$ are shown in Figure 4c and Figure 4d (respectively), as well as for the thermoelastic cell problem depicted in Figure 4e. Black lines identify the undeformed configuration. Note that, in this particular case, there is no need to solve numerically for $\chi_{h}^{22}$ and $\xi_{h}^{22}$ since the 90° rotational symmetry of the unit cell can be invoked.

Once all the cell problems are solved, we can compute the localization tensors $a_{\alpha \beta \gamma \delta }^{*}$, $b_{\alpha \beta \gamma \delta }^{*}$ and $t_{\alpha \beta \gamma \delta }^{*}$ given by (34) and thus all the homogenized properties (40). Finally, solving the linear system (42), we can compute the effective thermal expansion tensor $\alpha_{\gamma \delta }^{*}$ and the effective thermal-induced curvature tensor $\kappa_{\gamma \delta }^{*}$.

**Figure 5:**
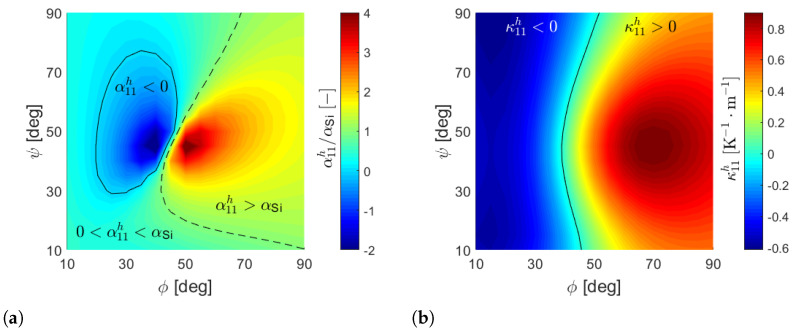
Contours of (**a**) $\alpha_{11}^{h}/\alpha_{\mathrm{Si}}$ and (**b**) $\kappa_{11}^{h}$ as a function of ϕ and ψ in the case $h_{1}=10h_{2}=10\;$µm, d=90µm and $t_{\mathrm{ext}}=t_{\mathrm{int}}=2\;$µm.

Figure 5a shows the contour of the back-scaled homogenized CTE $\alpha_{11}^{h}=\alpha_{11}^{*}$, normalized with respect to the CTE of silicon $\alpha_{\mathrm{Si}}$, as a function of ϕ and ψ. As is possible to observe, a proper selection of the inclination angles allows us to obtain a positive equivalent CTE $\alpha_{11}^{h}$ lower or larger than the one of silicon, or even negative. The black continuos line corresponds to $\alpha_{11}^{h}=0$, while the dashed one corresponds to $\alpha_{11}^{h}=\alpha_{\mathrm{Si}}$.

The back-scaled thermal-induced curvature $\kappa_{11}^{h}=\epsilon^{-1}\kappa_{11}^{*}$ is shown in Figure 5b as a function of ϕ and ψ. For high values of the angle ϕ, the cell exhibits a global downward deflection in the $x_{1}$-direction ($\kappa_{11}^{h}>0$) when heated, as one would reasonably expect from a layered material, with the top layer having a greater CTE than the bottom one. However, for low values of ϕ, the sign of the effective curvature changes and one obtains a global upward deflection for a positive temperature variation. The black line corresponds to $\kappa_{11}^{h}=0$.

Since a 90° rotation of the unit cell coincides with the cell that one obtains by swapping ϕ and ψ, we obtain $\alpha_{22}^{h}\left(\phi ,\psi \right)=\alpha_{11}^{h}\left(\psi ,\phi \right)$ and $\kappa_{22}^{h}\left(\phi ,\psi \right)=\kappa_{11}^{h}\left(\psi ,\phi \right)$. Therefore, the contours of $\alpha_{22}^{h}$ and $\kappa_{22}^{h}$ are coincident with those shown in Figure 5 but mirrored with respect to the bisector ϕ=ψ.

### 5.2. Convergence of the Homogenization Method

**Figure 6:**
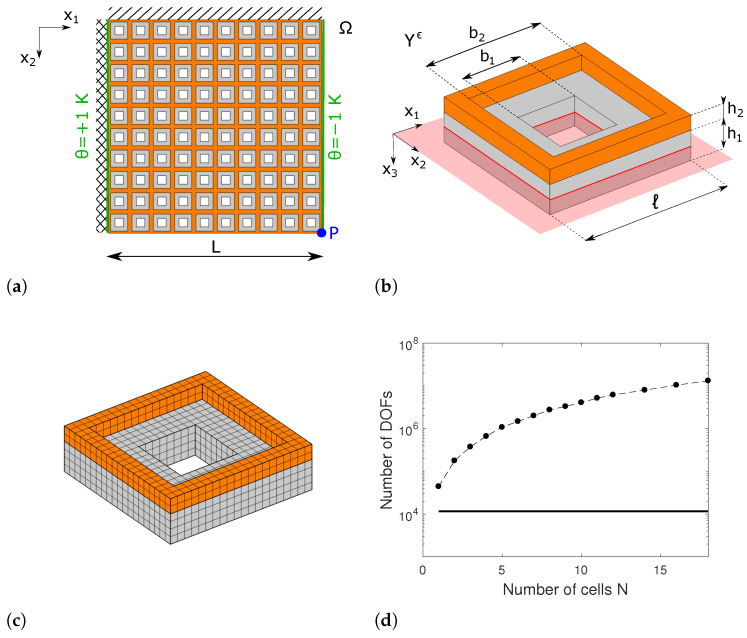
(**a**) Top view of the metamaterial plate; (**b**) geometry of the unit cell; (**c**) mesh employed for each cell; (**d**) comparison between DOFs of the real (markers) and homogenized (continuous) model against the number of cells *N*.

The purpose of this second example is to verify numerically the convergence of the homogenization technique, as ϵ→0, to the solution of the full numerical simulation on the actual geometry. The complex star-shaped geometry depicted in Figure 3 is not suitable to perform such a study since a very fine mesh is required to correctly reproduce its deformation and, thus, the numerical analyses with many cells would be computationally prohibitive. Therefore, we assess the convergence of the homogenization scheme with reference to another problem illustrated in Figure 6a. We consider a square plate of side L=1000µm clamped on the left edge, simply supported on the top one and free on the two remaining sides. On the left and right edges, a temperature variation θ=1 K and θ=−1 K (respectively) is prescribed to obtain a non-uniform temperature field in the metaplate.

The plate is the N×N, with N∈N, repetition of the unit cell shown in Figure 6b. The latter has an in-plane dimension ℓ=L/N and is composed of two layers, having heights $h_{1}=\ell /5$ and $h_{2}=\ell /10$, made, respectively, of silicon (in grey) and nickel (in orange). The two layers are perforated with square holes of sides $b_{1}=2\ell /5$ and $b_{2}=4\ell /5$. The reference mid-surface, depicted in red in the same figure, coincides with the mid-surface of the silicon layer. Note that, for this example, the effective CTE of the metaplate is expected to be positive. However, this is not relevant to the purposes of the convergence study.

Keeping fixed the global dimension *L* of the plate and all the boundary conditions, we compute the metamaterial response by varying the number of cells *N* on each side. In this way, for increasing values of *N*, we are numerically performing the limit ϵ=ℓ/L=1/N→0.

Figure 6c shows the mesh employed for the finite element discretization of a single unit cell of the metamaterial plate, which consists of quadratic serendipity hexahedral elements. Despite the mesh being quite coarse, the number of degrees of freedom (DOFs) for the whole problem grows rapidly with the number of cells *N*, as shown in the semilogarithmic plot in Figure 6d with markers. This shows clearly that it would be computationally prohibitive to deal with a large number of unit cells with complex geometry that requires a fine mesh, such as the one considered in Section 5.1. In the same plot, the continuous line indicates the number of DOFs employed to solve the homogenized problem, which is independent of *N*, and assesses the advantage of asymptotic homogenization in terms of computational cost.

**Figure 7:**
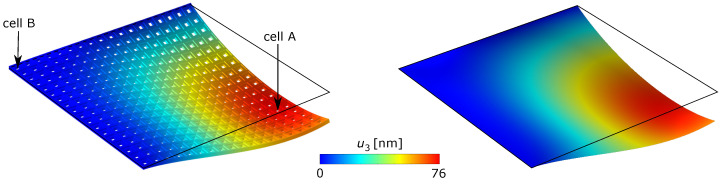
Contour on the deformed shape of the out-of-plane displacement component for the real metamaterial (**left**) and the homogenized plate (**right**) for N=18.

A first comparison between the real metamaterial and the homogenized plate can be performed on the displacement field. Figure 7 compares the out-of-plane displacement obtained by solving the real three-dimensional problem (left) and the equivalent plate (right) with eighteen cells per side, showing a good agreement between the two solutions. Black lines identify the undeformed reference mid-surface.

**Figure 8:**
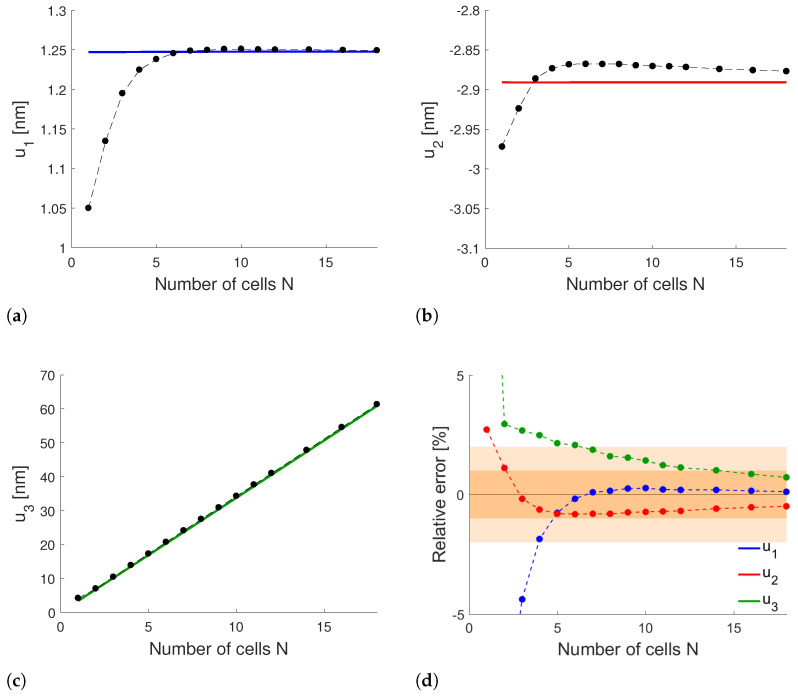
Comparison between real (dashed with markers) and homogenized (continuous) mid-surface displacement in point P against *N*: (**a**) $u_{1}$; (**b**) $u_{2}$; and (**c**) $u_{3}$. (**d**) Relative error between real and homogenized displacement components.

We also compute the in-plane displacement components $u_{1},u_{2}$ and the out-of-plane component $u_{3}$ of the plate mid-surface in point P, indicated in Figure 6a with a blue circle, for different values of *N*. Figure 8a, Figure 8b and Figure 8c compare the mid-plane displacement obtained from the solution of the real three-dimensional problem (dashed lines with markers) with that obtained from the homogenized plate (continuous line) through (43). The corresponding relative errors, shown in Figure 8d, tend to zero as *N* increases, showing the convergence of the real solution towards the homogenized one. The convergence is faster for $u_{1}$ (in blue) than $u_{2}$ (in red) and $u_{3}$ (in green). In particular, the displacement field is evaluated with an error already lower than 2% for N=7 cells (light orange region) and less than 1% for N≥16 (dark orange region), assessing, therefore, the accuracy of the method even with a limited number of cells.

**Figure 9:**
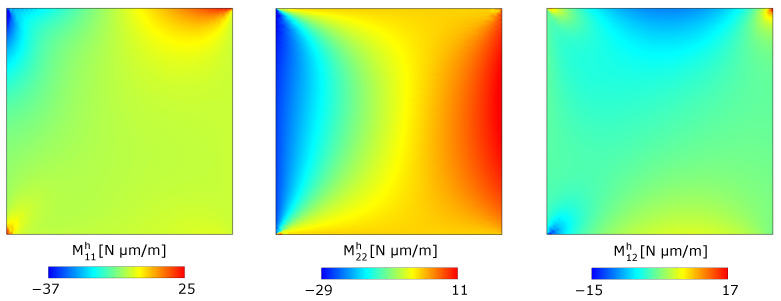
Contours of the back-scaled homogenized plate moments for N=18 (**a**) $M_{11}^{h}$; (**b**) $M_{22}^{h}$; and (**c**) $M_{12}^{h}$.

Asymptotic homogenization also allows for the computation of the state of stress within the periodic media. The solution of the homogenized problem provides the back-scaled effective plate membrane forces $N_{\alpha \beta }^{h}$ and moments $M_{\alpha \beta }^{h}$. The latter are shown in the contours of Figure 9 in the case N=18. However, these generalized plate stresses do not allow the direct identification of the stress distribution within the unit cells, since they are the resultant forces and moments of such stresses.

**Figure 10:**
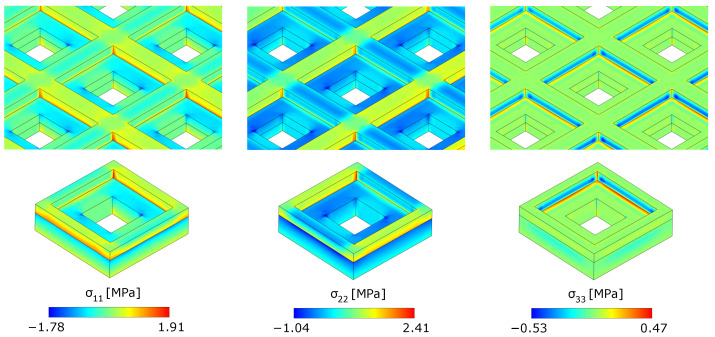
Contours of the stresses $\sigma_{11}$, $\sigma_{22}$ and $\sigma_{33}$ obtained from the real problem (top row) and those reconstructed through homogenization (bottom row) for the cell A.

The reconstruction of the local stress state $\sigma_{ij}^{h}$ within each unit cell can be performed exploiting Equation (44). For example, we consider the unit cell A, located in the point of maximum deflection of the metaplate, which is indicated with a black arrow in Figure 7. The stress components $\sigma_{11}$, $\sigma_{22}$ and $\sigma_{33}$ obtained through the numerical solution of the real three-dimensional problem, shown in the top row of Figure 10, are compared with those reconstructed through homogenization (bottom row). A satisfactory agreement can be observed.

**Figure 11:**
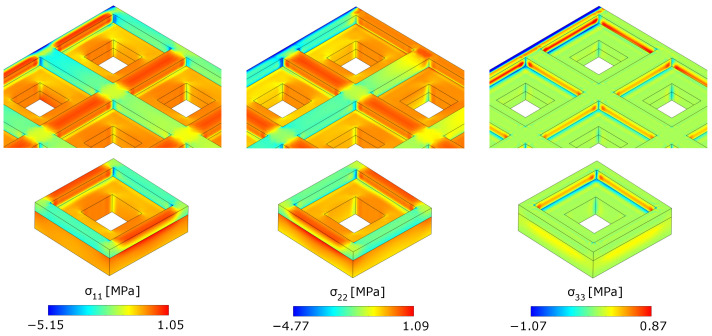
Contours of the stresses $\sigma_{11}$, $\sigma_{22}$ and $\sigma_{33}$ obtained from the real problem (top row) and those reconstructed through homogenization (bottom row) for the cell B.

It should be noted that the local stress fields reconstructed through homogenization are reliable only for unit cells sufficiently far away from the boundaries of the metamaterial. In fact, the stress concentration tensors (34) are $\hat{Y}$-periodic with respect $y_{1}$ and $y_{2}$ in the unit cell and, thus, cannot account for boundary effects. This can be clearly observed if one considers the cell B located at the top-left corner of the metamaterial plate, indicated in Figure 7 with a black arrow, where the left edge is clamped while the top one is simply supported. The real stress components, shown in the first row of Figure 11, exhibit a concentration on their boundaries that is not captured by the reconstructed stress through homogenization (same figure, bottom row).

## 6. Discussion

Standard asymptotic methods for the homogenization of thermoelastic solid metamaterials, as in [15], require that the dimension of the problem and of the periodicity match. If not, as in the case considered in this work of a three-dimensional body characterized by the two-dimensional repetition of a unit cell, only an in-plane characterization of the homogenized properties can be pursued. Under all the hypotheses assumed in Section 2, we extend the approach proposed in [26] and include thermal effects for the homogenization of a three-dimensional metamaterial endowed with a two-dimensional periodicity into an equivalent homogenous plate. The equivalent properties are defined on the mesoscale, in the sense of classical Cauchy continuum mechanics. The actual microstructure of the material, i.e., the dimensions of the unit cell, can be scaled down to tenths of a micrometre, but is still in the domain of validity of continuum mechanics. The effects arising at the nanoscale [29] are outside the aim of the present work.

The main finding of the proposed techniques is the numerical evaluation, through the solution of cell problems, of the homogenized properties of the equivalent plate. In particular, it is possible to characterize the effective (i) membrane, bending and coupling stiffnesses, (ii) thermal membrane forces and moments, (iii) thermal expansion tensor, (iv) thermal-induced curvature tensor and (v) thermal conductivity tensor. As shown with the numerical example of Section 5.1, this makes the homogenization method suitable to perform parametric studies useful for the design of metamaterials with programmable thermal properties including negative CTE.

When the unit cell geometry is different in the two in-plane directions, the equivalent thermal conductivity is anisotropic and other unusual properties could be obtained. Even if this possibility is not explored in the present work, anisotropic thermal properties could allow obtaining thermal cloaking, heat concentration or deviation [30].

Secondly, as discussed in Section 5.2, homogenization could be effectively employed to reduce the computational burden of numerical analyses, especially for metaplates with a large number of cells or complex microstructure. The homogenized displacement field is in good agreement with those computed by solving the real three-dimensional problem. A satisfactory agreement can also be observed in the local stress reconstruction, except for the unit cells close to the boundary, where the lack of periodicity does not allow predicting the real stress distribution [31]. This is a common feature of homogenization methods and it is still an open problem in the literature.

Regarding the limitations of the proposed homogenization technique, in this work we do not consider the transient effect of metamaterial plates. This limitation could be overcome by performing the asymptotic study starting from the fully coupled dynamic equation of thermoelasticity. This extension will be pursued elsewhere.

The most critical hypothesis of the developed homogenization procedure is the assumption of temperature-independent material parameters. For some applications, like in the MEMS field, the dependence of stiffness and the CTE on temperature becomes relevant and cannot be neglected. In such a case, the asymptotic expansion of the temperature field will enter into the material properties which need to be developed, as well, in series with respect to the small parameter ϵ, and the homogenization procedure will become more involved.

## 7. Conclusions

In this work, we study the steady-state thermoelastic problem for a three-dimensional periodic medium that is endowed with an in-plane two-dimensional periodicity. In the hypothesis of small transverse thickness $h^{\epsilon }$ and in-plane dimension *ℓ* of the single unit cell with respect to the global size *L* of the metamaterial, we fixed the ratio $h^{\epsilon }/\ell$ and performed an asymptotic analysis as ℓ/L→0.

We extended an already existing approach in the literature, which accounts only for a purely elastic problem, by introducing in the model the temperature field, which can be macroscopically varying.

The asymptotic homogenization technique thus obtained allows for the description of the three-dimensional metamaterial as that of an equivalent homogenous plate. In particular, the method provides the plate effective thermal expansion tensor and the thermal-induced curvature tensor, which, in general, are non-zero due to the transverse heterogeneity of the unit cell.

With reference to a complex star-shaped unit cell geometry, we show how homogenization can be exploited to perform parametric studies and that it can capture exotic behaviour such as negative thermal expansion and tunable thermal-induced curvature.

Finally, with reference to a square plate with inhomogeneous kinematic boundary conditions and a non-uniform temperature field, we discuss numerically the convergence of the real solution, as the number of cells increases, towards the homogenized one. The results obtained confirm the good accuracy of the homogenized solution, both in terms of displacement and stress, with a strong reduction in the computational cost.

## Figures and Tables

**Table 1:** Material properties at 20 °C.

Material	*E* [GPa]	ν [−]	α [ppm/K]
Silicon	160	0.22	2.6
Nickel	200	0.29	12.6

## Data Availability

The original contributions presented in the study are included in the article, further inquiries can be directed to the corresponding author.

## References

[1] Mary T.A., Evans J.S.O., Vogt T., Sleight A.W. (1996). Negative Thermal Expansion from 0.3 to 1050 Kelvin in ZrW_2_O_8_. Science.

[2] Khosrovani N., Sleight A., Vogt T. (1997). Structure of ZrV_2_O_7_ from −263 to 470 °C. J. Solid State Chem..

[3] Wang K., Chen J., Han Z., Wei K., Yang X., Wang Z., Fang D. (2022). Synergistically program thermal expansional and mechanical performances in 3D metamaterials: Design-Architecture-Performance. J. Mech. Phys. Solids.

[4] Dubey D., Mirhakimi A.S., Elbestawi M.A. (2024). Negative Thermal Expansion Metamaterials: A Review of Design, Fabrication, and Applications. J. Manuf. Mater. Process..

[5] Wu L., Li B., Zhou J. (2016). Isotropic Negative Thermal Expansion Metamaterials. ACS Appl. Mater. Interfaces.

[6] Lim T.C. (2005). Anisotropic and negative thermal expansion behavior in a cellular microstructure. J. Mater. Sci..

[7] Zhang Q., Sun Y. (2024). A series of auxetic metamaterials with negative thermal expansion based on L-shaped microstructures. Thin-Walled Struct..

[8] Vigna B., Ferrari P., Villa F.F., Lasalandra E., Zerbini S. (2022). Silicon Sensors and Actuators.

[9] Latella M., Faraci D., Comi C. A new metamaterial plate with tunable thermal expansion. Proceedings of the 9th European Congress on Computational Methods in Applied Sciences and Engineering.

[10] Bensoussan A., Lions J., Papanicolaou G. (1978). Asymptotic Analysis for Periodic Structures.

[11] Bakhvalov N., Panasenko G. (1989). Homogenisation: Averaging Processes in Periodic Media.

[12] Auriault J.L., Bonnet G., Nous I., Nous O. (1985). Dynamique des composites elastiques periodiques. Arch. Mech..

[13] Comi C., Marigo J.J. (2020). Homogenization Approach and Bloch-Floquet Theory for Band-Gap Prediction in 2D Locally Resonant Metamaterials. J. Elast..

[14] Craster R.V., Kaplunov J., Pichugin A.V. (2010). High-frequency homogenization for periodic media. Proc. R. Soc. Math. Phys. Eng. Sci..

[15] Francfort G.A. (1983). Homogenization and Linear Thermoelasticity. SIAM J. Math. Anal..

[16] Del Toro R., De Bellis M.L., Vasta M., Bacigalupo A. (2024). Multifield asymptotic homogenization for periodic materials in non-standard thermoelasticity. Int. J. Mech. Sci..

[17] Auriault J., Boutin C., Geindreau C. (2009). Homogenization of Coupled Phenomena in Heterogenous Media.

[18] Duvaut G. (1977). Comportement macroscopique d’une plaque perforee periodiquement. Singular Perturbations and Boundary Layer Theory. Lecture Notes in Mathematics.

[19] Lewiński T. (1991). Effective models of composite periodic plates-III. Two-dimensional approaches. Int. J. Solids Struct..

[20] Faraci D., Comi C., Marigo J.J. (2022). Band Gaps in Metamaterial Plates: Asymptotic Homogenization and Bloch-Floquet Approaches. J. Elast..

[21] Duvaut G. (1978). Homogeneisation des plaques a structure periodique en theorie non lineaire de von Karman. Lect. Notes Math..

[22] Faraci D., Comi C. Nonlinear behaviour and homogenization of metaplates. Proceedings of the X ECCOMAS Thematic Conference on Smart Structures and Materials.

[23] Caillerie D. (1982). Plaques élastiques minces à structure périodique de période et d’épaisseur comparables. C. R. Acad. Sci. Paris.

[24] Lewiński T. (1991). Effective models of composite periodic plates-I. Asymptotic solution. Int. J. Solids Struct..

[25] Caillerie D., Nedelec J.C. (1984). Thin elastic and periodic plates. Math. Methods Appl. Sci..

[26] Lewiński T., Telega J.J. (1999). Plates, Laminates and Shells: Asymptotic Analysis and Homogenization.

[27] Lemaitre J., Chaboche J.L. (1990). Mechanics of Solid Materials.

[28] Holzapfel G.A. (2000). Nonlinear Solid Mechanics.

[29] Hernández-Acosta M., Martines-Arano H., Soto-Ruvalcaba L., Martínez-González C., Martínez-Gutiérrez H., Torres-Torres C. (2020). Fractional thermal transport and twisted light induced by an optical two-wave mixing in single-wall carbon nanotubes. Int. J. Therm. Sci..

[30] Li W., Sigmund O., Zhang X. (2024). Analytical realization of complex thermal meta-devices. Nat. Commun..

[31] Dumontet H. (1986). Study of a boundary layer problem in elastic composite materials. Math. Model. Numer. Anal..

